# Greenstone artifacts in pre-Columbian Costa Rica: from raw material to local and interregional exchanges

**DOI:** 10.1038/s41598-026-52689-z

**Published:** 2026-05-17

**Authors:** Camila Hernández-Murillo, Sergio García Piedra, Silvia Salgado, Cleria Ruiz Torres, Mavis L. Montero, Robert Dinnebier, Matthieu Ménager

**Affiliations:** 1 IMBE, Avignon Université, Aix Marseille Université, CNRS, IRD, Avignon, France; 2 Universidad de Costa Rica, San José, Costa Rica; 3 Centre d’Études Mexicaines et Centraméricaines, UMIFRE 16, MEAE, CNRS, Mexico city, Mexico; 4 Museo del Jade y de la Cultura Precolombina, San Jose, Costa Rica; 5 Center for Research in Materials Science and Engineering, University of Costa Rica, San José, Costa Rica; 6 Museo Nacional de Costa Rica, San José, Costa Rica; 7 Max Planck Institute for Solid State Research, Stuttgart, Germany

**Keywords:** pre-Columbian Costa Rica, Greenstones, Archaeometry, Long-distance exchange, Jadeite, Chemistry, Solid Earth sciences

## Abstract

Understanding the distribution pattern and relative abundance of the different greenstones used by pre-Columbian societies in the current territory of Costa Rica provides valuable insights into ancient exchange networks. This study examines the role of local and interregional exchange systems involving both locally sourced and allochthonous greenstones in the Greater Nicoya and the Central Caribbean regions. Non-invasive infrared spectroscopy was applied to identify the main mineral phases in 926 objects from 54 archaeological sites, and chemometric analyses were used to distinguish compositional groups and to visualize their spatial distribution. X-ray diffraction was additionally performed on five representative samples, one from each of the most frequent compositional groups, to complement the spectroscopic analysis. The results reveal that each region displays distinct patterns of greenstone use, reflecting differences in access to materials and participation in exchange networks. Data suggests that locally available minerals, such as serpentine, tremolite-actinolite, quartz and other siliceous rocks, were obtained through short-range procurement systems whereas allochthonous allochthonous jadeitite and albitite were involved in long-distance interregional exchange. These findings highlight the coexistence of regional and long-distance networks and demonstrate the key role of jadeite in linking distinct lithic traditions within Costa Rica and beyond.

**Supplementary Information:**

The online version contains supplementary material available at 10.1038/s41598-026-52689-z.

## Introduction

**Fig. 1:**
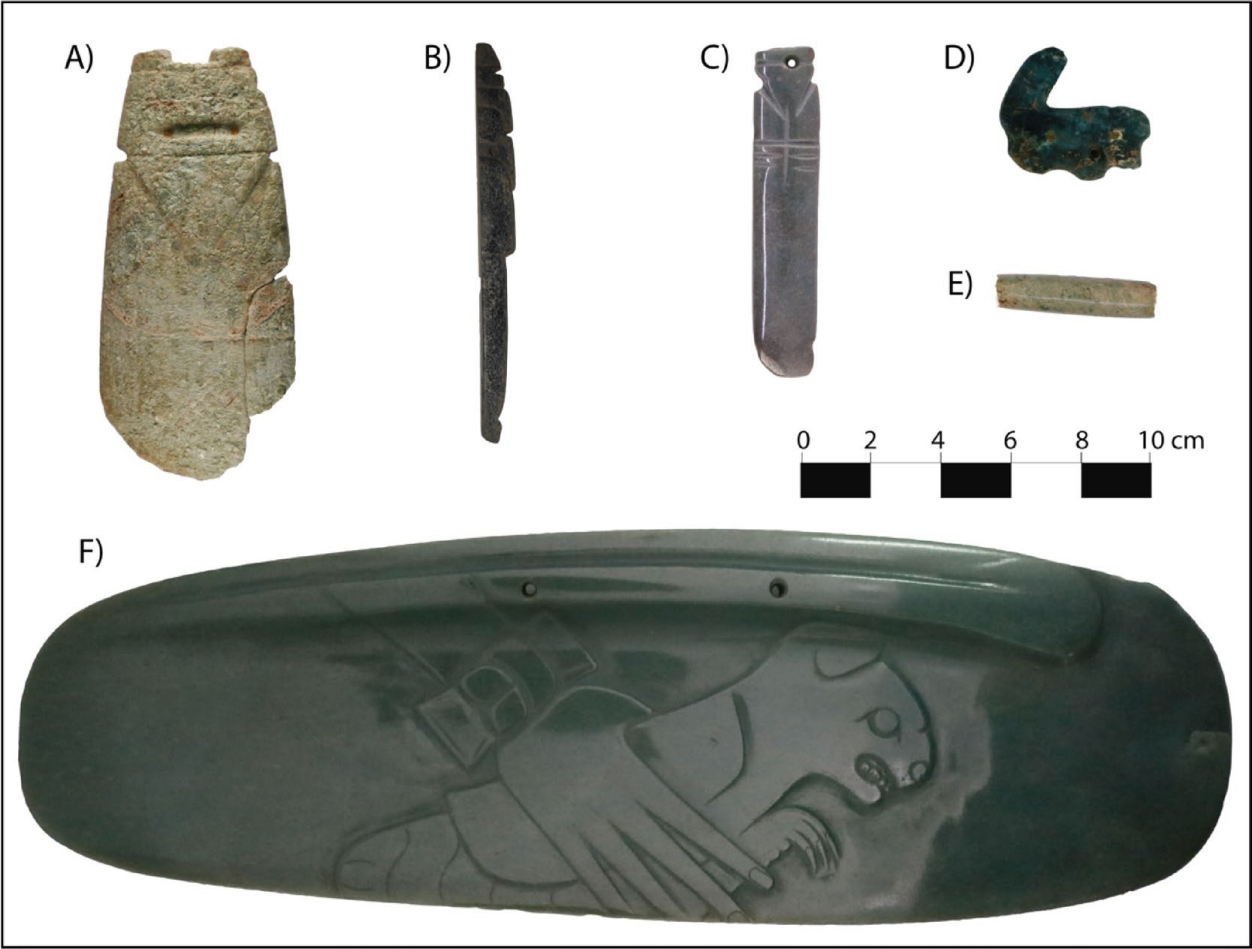
Examples of jade and greenstone artifacts from the following archaeological sites in Costa Rica and its corresponding approximate dates by association with ceramic type: (**A**) Rempujo (Artifact 52, 500 B.C. - A.D. 300), (**B**) Loma Corral (Artifact 82, 500 B.C. - A.D. 300), (**C**) Fina Linares (Artifact 125, A.D. 300 - A.D. 800), (**D**) Ballena (Artifact 106, 500 B.C. - A.D. 300), (**E**) La Fábrica (Artifact 160, 300 B.C. - A.D. 1500) and (**F**) Talamanca de Tibás (Artifact 36, A.D. 300 - A.D. 800).

More than 2500 years ago, pre-Columbian groups living in the northern Chibcha area, in territory that currently corresponds to Costa Rica, developed a high level of technical skills and cultural sophistication. Artisans worked a variety of hard stones, including a wide range of greenstones, to produce finely elaborated pendants, beads, celts, metates, sculptures, petroglyphs and ceremonial axes^1–7^ (cf. Fig. 1). The abundance of these stone objects in pre-Columbian contexts reflects a remarkable level of lapidary techniques, with a high degree of innovation and technical mastery, a good territory knowledge, control (for stone deposits) and sometimes the presence of long-distance networks (for jadeitite artifacts). The persistence and refinement of this lapidary production over more than 1000 years also shows a great transmission of this specialized knowledge across generations. Fine stone artifacts were mainly found in funerary contexts where they were deposited as offerings, presumably used during the individual’s lifetime. Much of this lapidary production was more likely integrated with religious practices, elite and group identities and interregional relationships and trades, following the emergence and evolution of the social and ideological complexity in the area.

In archaeological literature, the term “greenstone” refers to a broad category of polished lithic materials of symbolic and social significance and does not necessarily imply a strictly green coloration. Many lapidary artifacts produced in pre-Columbian Costa Rica were crafted using different types of greenstones, extracted from local deposits or long-distance trade (jadeitite, albitite). Such types of greenstone artifacts included zoomorphic, chimeric, anthropomorphic, or simple pendants, tubular and globular beads, ceremonial mass heads, celts and axes^2,8,9^. These artifacts were found in archeological contexts from the geographic area corresponding to the northern two-thirds of the current territory of Costa Rica^1,7,10–12^, from the Talamanca mountain range to the north of the Guanacaste province, at the northern limit of the Isthmo-Colombian cultural region^13^.

Within this lapidary production, the hachoïd-shaped pendants, also called “dios hacha” or axe gods^8^ are maybe the most characteristic archeological goods from the area, and are a representative marker of this lapidary tradition, with a first occurrence around 500 ± 100 BCE (site of Huiscoyol). The earliest presence of jadeitite in an archeological context of the area (site of La Regla) more likely date from 300 ± 100 BCE^11^. These “dios hacha” were indeed found in funerary contexts from the sites of La Regla and Huiscoyol on the Nicoya peninsula and with associated carbon-14 (^14^C) dating from woods, human bones (La Regla: grave 2, 392 − 204 BCE; grave 4, 385 − 197 BCE and 401 − 20 BCE; grave 5, 392 − 204 BCE), carbonized material (Huiscoyol: grave 11, 390 − 202 BCE) and charcoal (Huiscoyol: grave 14, 409 − 211 BCE; operation 2, artifact 75, 733 − 397 BCE ND with a probability of 86.4% for the 546 − 397 BCE range). In Costa Rica, this tradition ended around A.D. 600, after which its relevance started to decline^5,14^. Although the earliest examples of so-called “dios hacha” pendants most likely emerged along the Pacific littoral of the Greater Nicoya region^4^, the tradition spread eastward into the Caribbean lowlands and, to a lesser extent, southeastward into the volcanic central highlands. As it travelled, local artisans reinterpreted the basic axe-blade characteristics to reflect their own iconographic repertoire.

Among all “dios hachas”, the avian-shaped pendants or “ave pico” have distinctive bird features, typically a beak and wings, in their upper section and an axe-shaped lower section. It is important to point out that similar avian-shaped axe pendants have been found in funerary offerings from the Middle Formative Period (900 − 400 B.C.) (i) in northern Honduras^15^, particularly at sites along the lower Ulúa River valley, (ii) in the Pacific coast of Guatemala, at the sites of Tak´alik Ab´aj site^16^, (iii) in Chiapas, in a cave at 50 miles from San Cristóbal de las Casas^17^, (iv) at Chalchuapa site in the Pacific coast of Salvador, (v) in the Yucatán peninsula^18^, notably at the Chaksinkin site (vi) at the Kaminaljuyu, Altun Ha and Pomona sites in the maya area^18^.

An important proportion of all these greenstone objects present in museums within Costa Rica and across the globe (United States, France, Sweden, Germany, etc.) were obtained through illicit excavation, meaning that hardly any information about their archeological contexts, depositional settings, and associated dating has been preserved. Moreover, just a few of these archeological sites have ever been analyzed in peer-reviewed studies. Indeed, most excavation reports leave the raw material unanalyzed and unidentified, with very limited data on dating^11^.

Moreover, many sites excavated during the 1970 –1990 s still rely on incomplete inventories, and potential reclassified contexts were never entered into modern catalogues or artifact databases. Adding to the confusion, identification numbers within a site were often re-used, assigned by raw material, field season, or cultural trait, which greatly complicates tracing individual artifacts across records.

**Fig. 2:**
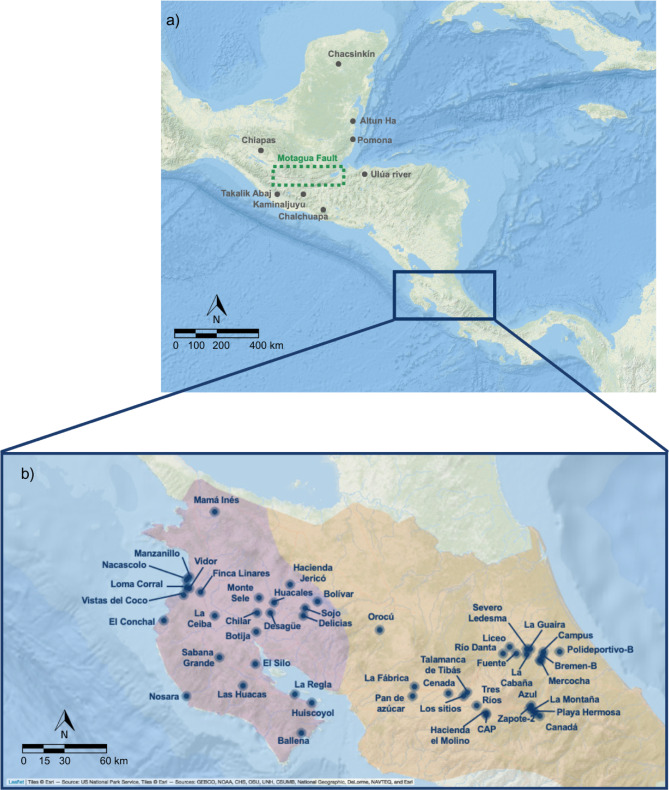
Maps showing (**a**) the Maya region, Central America and parts of the Caribbean region, relevant sites from the maya area are marked in grey. The green dotted rectangle marks the approximate location of the Motagua Fault Zone. The blue rectangle marks the location of the area of interest within Costa Rica, which is shown in blown up (**b**). The Greater Nicoya archaeological region is shaded in pale purple and the Central Caribbean archaeological region is shaded in pale orange, additionally archaeological sites included in this study are marked by blue dots. Base map: Esri WorldPhysical © Esri, USGS, NOAA; Esri Ocean Basemap © Esri, USGS, NOAA.

The classification of artifacts as “jade” continues to suffer from frequent mistakes, and a wide array of visually similar stones are routinely misidentified. Some researchers often label non-jade materials, green jaspers and serpentinites as jade, while in other cases jadeitite objects remain undetected due to inadequate identification. Polished surfaces offer few clues: once burnished, both quartz and jade exhibit the same aspect, rendering visual inspection deeply unreliable, even by geologists. Definitive identification therefore requires physicochemical techniques, like infrared (IR) or Raman spectroscopy, or portable X-ray diffraction (XRD) to allow a non-destructive identification of jadeitite from similar-looking materials.

Although many archaeological investigations have revealed contexts with jadeitite artifacts, only few applied such types of analyses, leaving significant gaps in the regional narrative and understanding of greenstone symbolic and economic importance.

Previous archaeometric studies on Costa Rican jade, including neutron-activation analysis, X-ray diffraction, and portable infrared and Raman spectroscopy, have consistently identified jadeite as the dominant phase^2,9,19^. However, other minerals (e.g. quartz, green jasper, omphacite, prehnite, feldspars, albite, with minor analcime, paragonite and muscovite) were also detected, underscoring the mineralogical diversity of the assemblage.

However, a large proportion of the artifacts examined in previous research came from unprovenanced collections that museums had acquired through donations purchases, voluntary restitution and confiscation. This most likely led to preferential acquisition of the more prestigious artifacts and semi-precious stones, of particular interest for a museum and its visitorship, leading to an overrepresentation of jadeitite over other stones. This introduces a critical constraint in the archaeological interpretation of data, with no possibility of local or inter regional comparison.

A detailed characterization of the raw materials is thus essential for understanding the mode of production, circulation and symbolism of these greenstone artifacts from sites in the north of Guanacaste and the Costa Rican Caribbean region. Indeed, the major objective of the paper is to clearly show that both local and long-distance exchange networks had key roles in the millenary lithic tradition shared from the Greater Nicoya to the Caribbean watershed.

Furthermore, this article aims to understand the relative contribution of locally extracted green stones and imported raw jadeitite blocks in the pre-Columbian Costa Rica area using archaeometry. It aims to reconstruct the ancient pathways in order to clarify how short-range procurement and long-distance trade functioned. We want to show the differences of greenstone uses between two important archeological areas of the Isthmo-Columbian region: Greater Nicoya and Central Caribbean in terms of jadeitite control by pre-Columbian societies, connection and raw material exchanges.

To quantify these flows, we merged the Museo Nacional de Costa Rica (MNCR) and the Carnegie Museum of Natural History (CMNH) inventories, more than 900 artifacts in over 7 years of analysis, into a unified database that records artifactual, archeological and chemical data, revealing the scale of exchange and marked regional pattern undescribed until now. Analytically, surface roughness (linked to polishing techniques) was shown to strongly affect infrared spectra in our previous works^20^. By targeting microscopic zones of comparable roughness and applying multivariate statistics, we achieved a robust distinction between imported jadeitites and local greenstones. We also used an exhaustive approach in the construction of the analytical corpus: every possible object with a certain or geo-referenced context was analyzed, regardless of prestige or complexity. The resulting map exposes greatly different distribution patterns by archaeological zone, leading to a reassessment of both regional and long-distance exchange models. Ultimately, this multidisciplinary collaboration, between curators, archaeologists, archaeometrists and chemists, shows that the combination of suitable analytical tools, rigorous statistics and international cooperation is the only way to reconstruct the dynamics of pre-Hispanic green-stone circulation and the diverse social meanings linked to these materials.

## Materials and methods

### Sample description

A total of 926 greenstone objects were analyzed with infrared spectroscopy. A wide array of object types was included, for example: beads, hachöid pendants, fragments, plaques, zoomorphic pendants and blocks of raw material. The non-invasive analysis was performed on 479 objects from the collections at the Museo Nacional de Costa Rica, and 447 objects from the Las Huacas site that are part of the collections at the Carnegie Museum of Natural History (Pittsburgh, USA). The former collection was analyzed extensively, and all the artifacts that could be analyzed were included in the study. In the case of the latter, an extensive approach could not be used due to the size of the collection and due to time constraints, however, a representative sample was selected based on the archaeological information available on each object. Only very small beads and heavily eroded fragments were excluded because of the analytical limitations of the infrared spectrometer. Supplementary Table S1 includes the complete list of artifacts analyzed as well as a summary of the main minerals identified per site.

The objects included in this study come from 54 archaeological sites, distributed along the Central Caribbean (*n* = 27) and the Greater Nicoya (*n* = 27) archaeological regions (see Fig. 2), over the time period between approximately 500 B.C. to A.D. 1550.

The approximate volume of each artifact was calculated by multiplying height, length and width of each artifact.

We further analyzed 5 artifacts from the Carnegie Museum of Natural History using X-ray diffraction. Due to the sampling needed to perform powder X-ray diffraction, the samples included in this analysis were restricted to a very limited number and come from previously broken artifacts. The objects were selected based on the results of spectroscopic characterization, if possible, one sample from each of the most frequent materials was chosen. A small fragment (approximately 150 mm^3^) of each of the selected objects was sampled using a handheld rotary tool equipped with a diamond cutting disc. The artifacts sampled are: 2438 − 514, 2939 − 558, 2939 − 1067, 2438 − 519 and 2939 − 558. Additionally, a commercial jadeitite sample from Motagua (Guatemala) was included in the XRD analysis and was used as a jadeitite reference.

### Infrared analysis

A portable FT-IR spectrometer Bruker Optics Alpha with an external reflection module was used for recording the infrared spectra, for each spot 35 scans were carried out to improve signal-to-noise ratio, focusing in the spectral region between 398 and 4000 cm^ −1^. The spot size has a 3 mm diameter. The number of points analyzed for each sample varied between 2 and 10 spectra, depending on sample size and shape. Every spectrum was taken exclusively on polished areas of the samples. The analyses were performed in situ, at each of the museum’s facilities. Spectra were compared with reference spectra from the RRUFF database^21^(http://rruff.info/) and band assignment was performed using previously reported data^22–27^.

### Data pre-processing and statistical analysis of FT-IR spectra

A series of pre-processing procedures were applied to the spectroscopic data using R software and were chosen according to the spectral requirements: computing of mean spectra per sample, cropping (the range of the FT-IR spectra was cropped to 400–1500 cm^− 1^), Kubelka-Munk transformation to reduce influence of scattering effects, rubberband baseline correction, normalization of spectrum intensities and Savitzky-Golay smoothing interpolation. Statistical analysis and data pre-processing was performed using the computing environment R. Data formatting and figures were prepared using the collection of R packages: tidyverse^28^, hyperSpec^29^, mapview^30^ and ggplot2^31^. Principal component analysis was performed as an exploratory technique, using a dataset comprising the pre-treated mean spectra per sample and the intensity for the corresponding wavenumber in the selected spectral range (400–1500 cm^− 1^).

### Powder X-ray diffraction analysis

The powder X-ray diffraction analysis was performed in the Max Planck Institute for Solid State Research in Stuttgart. The selected samples were manually ground using an agate mortar. The resulting powders were sealed in 0.5 mm diameter borosilicate capillaries. Diffraction patterns were collected in Debye–Scherrer geometry using a STOE StadiP diffractometer equipped with Mo-K_α_ radiation (λ = 0.71073 Å), a Ge(111) monochromator, and a triple array of Mythen 1 K detectors. The patterns were recorded in a 2θ range from 2.0° to 120° with a total scan time of 1 h. The capillaries were continuously spun during the measurements.

Qualitative phase analysis and diffraction data pre-processing were performed using the QualX software^32^ and the POW_COD database, based on the Crystallography Open Database^33^. The specific COD reference files used are detailed in Fig. 5.

## Results and discussion

### Materials used as greenstones: identification and chemometrics

**Fig. 3:**
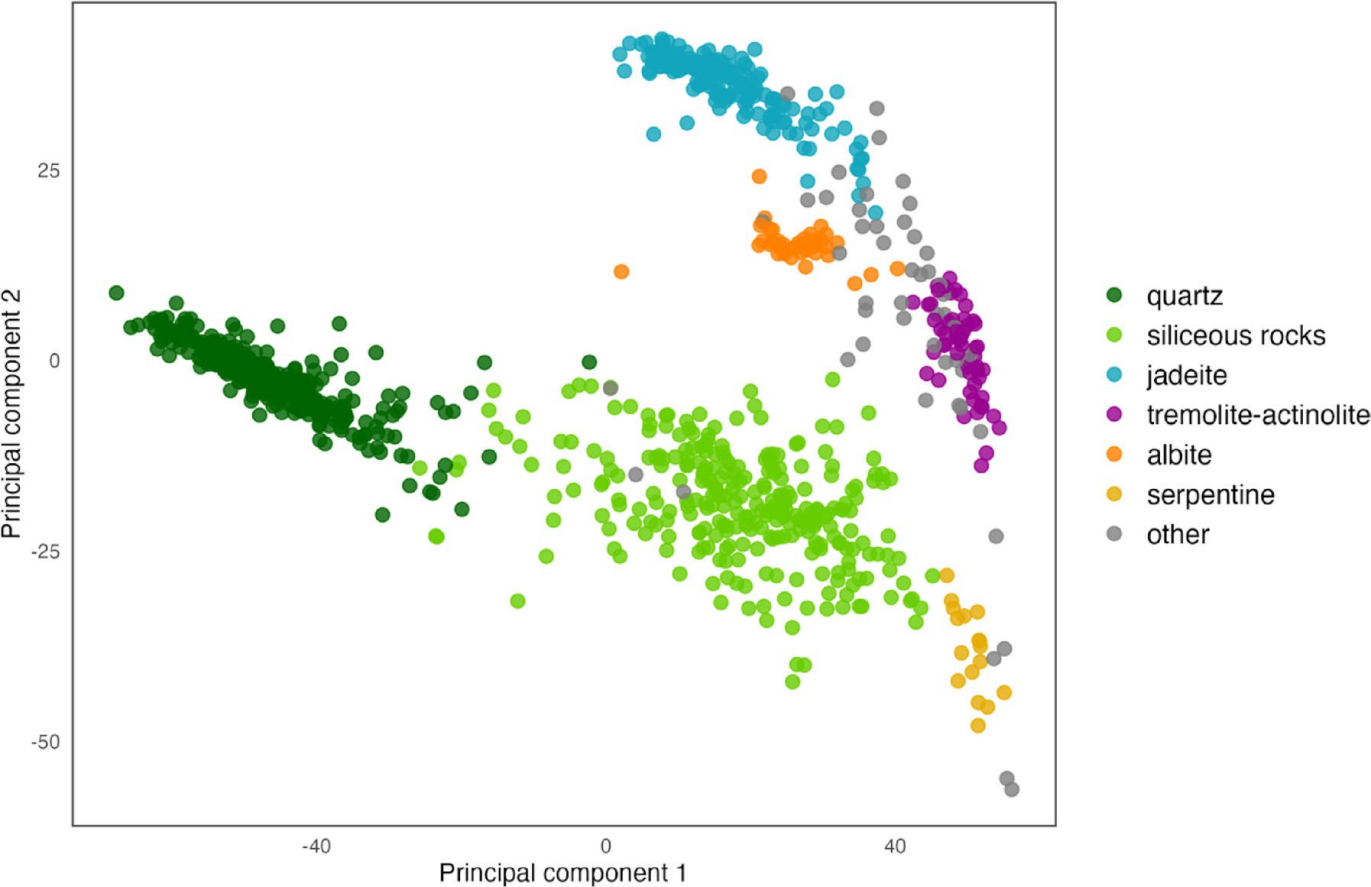
Scores (scatter plot) of the first two principal components for the pre-treated FT-IR spectra of the 926 greenstone objects analyzed. The colors were attributed to the distinct main minerals identified in the rocks.

FT-IR analysis allowed the non-invasive spectroscopic characterization of the greenstone objects. To better understand the mineralogical diversity of the samples, principal component analysis was performed on all pre-treated infrared spectra (see Fig. 3); it shows that six types of rocks were the most commonly used in pre-Columbian times. These rocks have different mineralogical compositions and the identified predominant constituent minerals were: quartz (dark green), jadeite (light blue), albite (orange), tremolite-actinolite (purple), serpentine (yellow) and some mixed silica minerals (siliceous rocks, light green). These six groups account for nearly 90% of the 926 objects included in this study. The term siliceous rocks used in this publication refers to rocks predominantly composed of micro- to cryptocrystalline silica of uncertain or mixed origin, which for example includes jasper and siliceous volcanoclastic rocks.

The dispersion of points within each group likely reflects subtle spectral variations, which can result from surface characteristics of the objects (e.g., roughness)^20^, grain size^34^, degree of crystallinity^35^, or the internal structural order of the material^36^. Additionally, variations in mineralogical composition, such as different proportions of secondary minerals or impurities, contribute to this scattering.

The two different types of rocks identified as polycrystalline quartz and siliceous rocks display characteristic SiO_2_ bands: a strong reflectance around 1100 –1050 cm^− 1^ due to the asymmetric stretching vibration of Si-O-Si, the characteristic double bands at 797 and 778 cm^− 1^ attributed to the symmetric stretching of Si-O-Si, and a band between 450 and 500 cm^− 1^ at for Si-O and O-Si-O bending^22^. The quartz objects present broader bands, in particular the signal attributed to asymmetric stretching vibration of the Si-O-Si at 1086 cm^− 1^; whereas in the siliceous rocks group this band is centered around 1075 cm^− 1^.

**Fig. 4:**
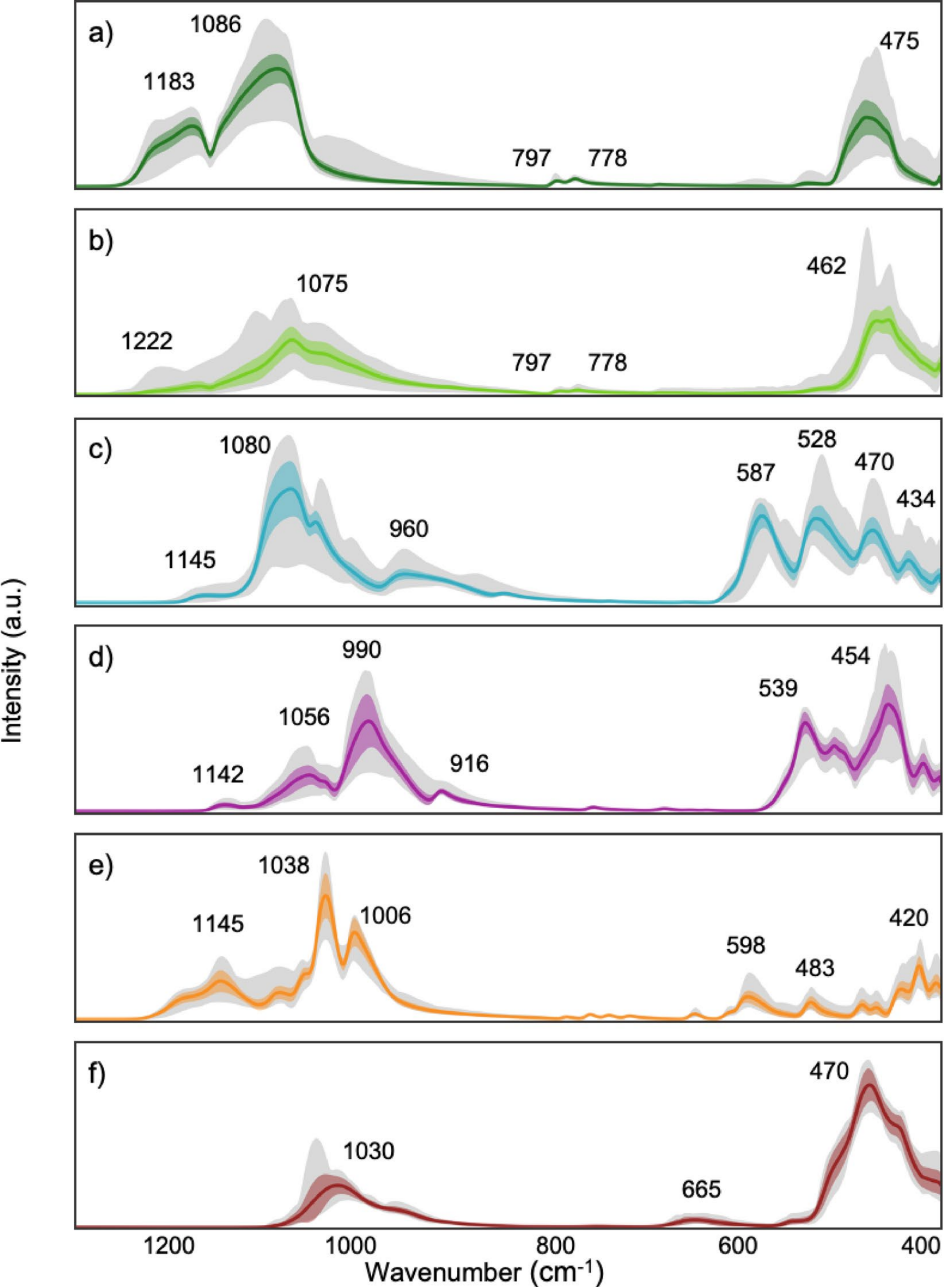
Mean infrared spectrum (dark line) for each of the 5 major compositional groups identified: (**a**) quartz, (**b**) siliceous rocks, (**c**) jadeite, (**d**) tremolite-actinolite, (**e**) albite, (**f**) serpentine. In each case, the light-colored areas represent the standard error over the spectrum while the grey areas represent the maximum and minimum values of the spectra.

As shown in Fig. 4, another characteristic from the quartz group is that it has a higher reflectance in the 1200 –900 cm^− 1^ range, in comparison to the other quartz-bearing rocks (siliceous rocks). The X-ray diffraction analysis of sample 2939 − 1067, from the quartz group, in agreement with the results from infrared spectroscopy, yielded the exclusive identification of quartz (Fig. 4-a).

The XRD pattern of the sample 2438 − 514, from the siliceous rocks group, matches with the phases: quartz, heulite and mordenite, two members of the zeolite group (Fig. 4-b). Mordenite was also detected in the infrared spectra thanks to the bands centered at: 1222, 1177, 1053 and 453 cm^− 1^. Heulandite characteristic bands also appeared at 1056 and 462 cm^− 1 23^. All of these bands are present, to a variable extent, in the spectra of samples from the siliceous rocks group. As shown in Fig. 4-b, their spectra show greater variability in the 1250 –1050 cm^− 1^ range and around 460 cm^− 1^ which might be linked to different proportions of these zeolites in the rocks. This also explains why the samples identified in this group show the greatest dispersion in the PCA score plot (Fig. 3).

**Fig. 5:**
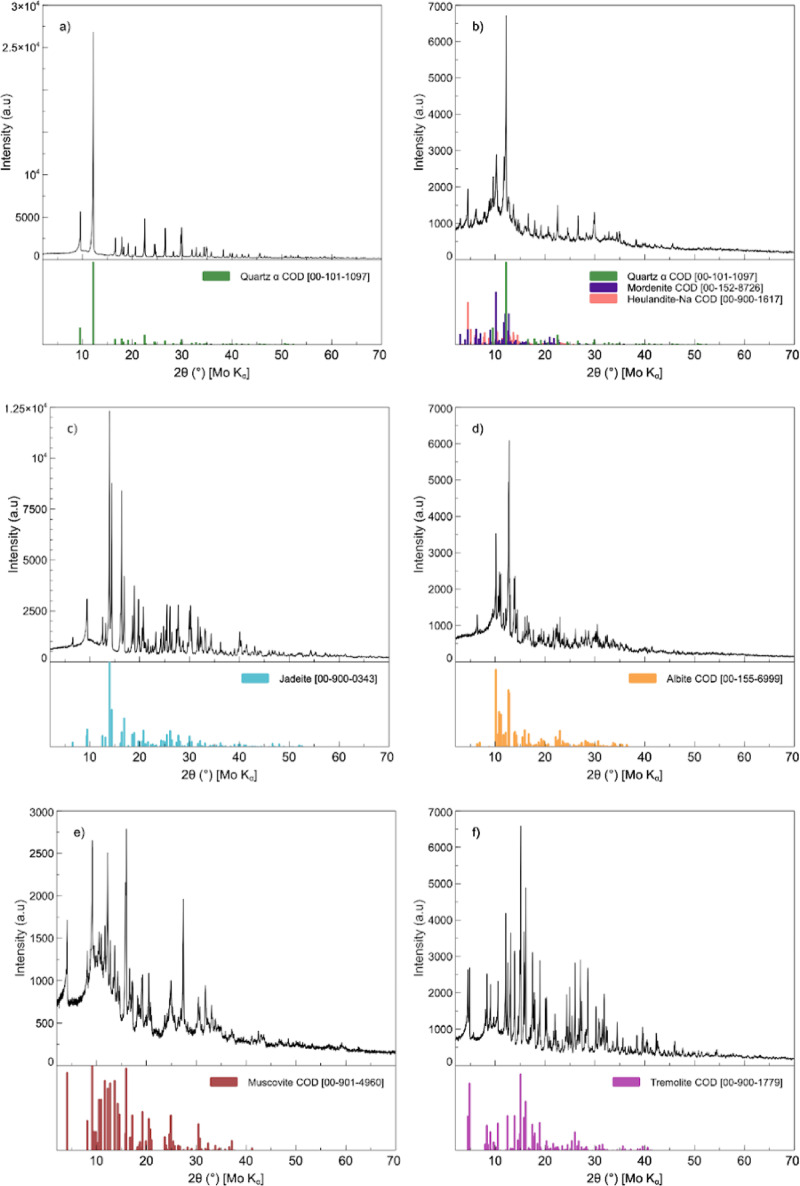
X-ray diffraction patterns with Mo source of selected greenstone objects from the collections of the CMNH and of a commercial jadeite sample with their respective reference peaks obtained from the Crystallography Database (COD): (**a**) 2939 − 1067, (**b**) 2438 − 514, (**c**) jadeite reference, (**d**) 2939 − 425, (**e**) 2939 − 558, (**f**) 2438 − 519.

A group of 165 objects were classified as jadeitite, a rock composed mainly of the mineral jadeite along with some of its common accessory minerals such as albite and omphacite. XRD analysis was performed on a jadeitite reference sample that displayed the same spectral features as the average spectra from this group: 1145 and 1080 cm^− 1^ for the asymmetric Si-O-Si stretching, 960 cm^− 1^ for the asymmetric stretching of Si-O, 665 and 477 cm^− 1^ for the Si-O-Si bending and SiO_4_ twisting at 434 and 587 cm^− 1 24^. As shown in Fig. 4-c, jadeite was the only mineral identified by XRD in the jadeitite reference sample. The mean infrared spectrum of this group, shown in Fig. 4-c, show significant variation between the minimum and maximum value of intensity, especially in the frequency range between 1110 –870 cm^− 1^ which can be related to different percentages of omphacite in the samples, whereas four small but well-defined peaks between 800 –700 cm^− 1^ are rather linked to the presence of albite in the rock. The identification of these associated minerals is to be expected and has been widely reported before, both in geological samples from the jadeitite source in the Motagua valley^37–39^ and in archaeological Mesoamerican objects^24,40,41^.

Furthermore, 57 objects were classified as tremolite-actinolite: both the group’s mean infrared spectra (Fig. 4-d) and the diffractogram of a representative sample form this group (Fig. 5f) display the characteristic features of the mineral tremolite. More specifically, the FTIR spectra show the distinct bands between 1200 –900 cm^− 1^ corresponding to the Si-O stretching vibrations, and the bands at 539 and 454 cm^− 1^ related to the O-Si-O bending^42^.

Regarding the group of 39 artifacts identified as albitite, their spectra show albite’s distinctive bands related to the asymmetric stretching of Si-O-Si (1145, 1109, 1038 and 1006 cm^− 1^), Al-SiO_4_ interactions (534 cm^− 1^), Si-O-Si bending (788 and 483 cm^− 1^)^43^. The small variation in intensity with respect to the mean spectrum (see Fig. 4-e) and the limited dispersion observed in the PCA plot (Fig. 3) suggest that this group has a relatively homogeneous composition. The diffraction analysis for sample 2939 − 425, from the albite showed the exclusive presence of albite diffraction peaks.

A set of 17 objects was identified as serpentinite, a rock formed mainly by minerals from the serpentine mineral subgroup. As seen in Fig. 4-f, the spectra assigned to this group show small variation with respect to the group’s mean spectrum, as well as its characteristic bands for Mg-O translation and SiO_4_ stretching at 470 cm^− 1^, Mg-OH bending at 665 cm^− 1^ and Si-O-Si stretching at 1030 cm^− 1 27^.

In addition to these predominant groups identified by infrared spectroscopy, there were others that were found less frequently, for instance: muscovite, prehnite, minerals other than albite from the feldspar group and from the chlorite group. This diverse group is denoted as “other” in Fig. 3. A detailed description of their spectral characteristics is described in Supplementary Table S2 available online.

Out of the 926 samples analyzed, in 21 samples it was not possible to achieve a concrete identification of the mineral composition, these samples are labeled as *other* in the PCA plot (Fig. 3).

It is also important to mention that there is a relevant number of objects that have a rough finish on their surface, and it is therefore impossible to apply the infrared spectroscopy characterization, this is the case for most sedimentary and igneous rocks, therefore these rocks types are not represented in these results. Another limitation intrinsic to the infrared spectroscopy technique by reflection is its limitation to identify accessory minerals in the rocks, the identification is restricted to major minerals as previously discussed in the literature. Nonetheless, the materials that were identified are of particular interest for understanding potential exchanges of raw materials, both foreign and local, as well as the knowledge and specialization involved in the craftsmanship of these lithic objects.

### Distribution of archaeological greenstones in Costa Rica

**Fig. 6:**
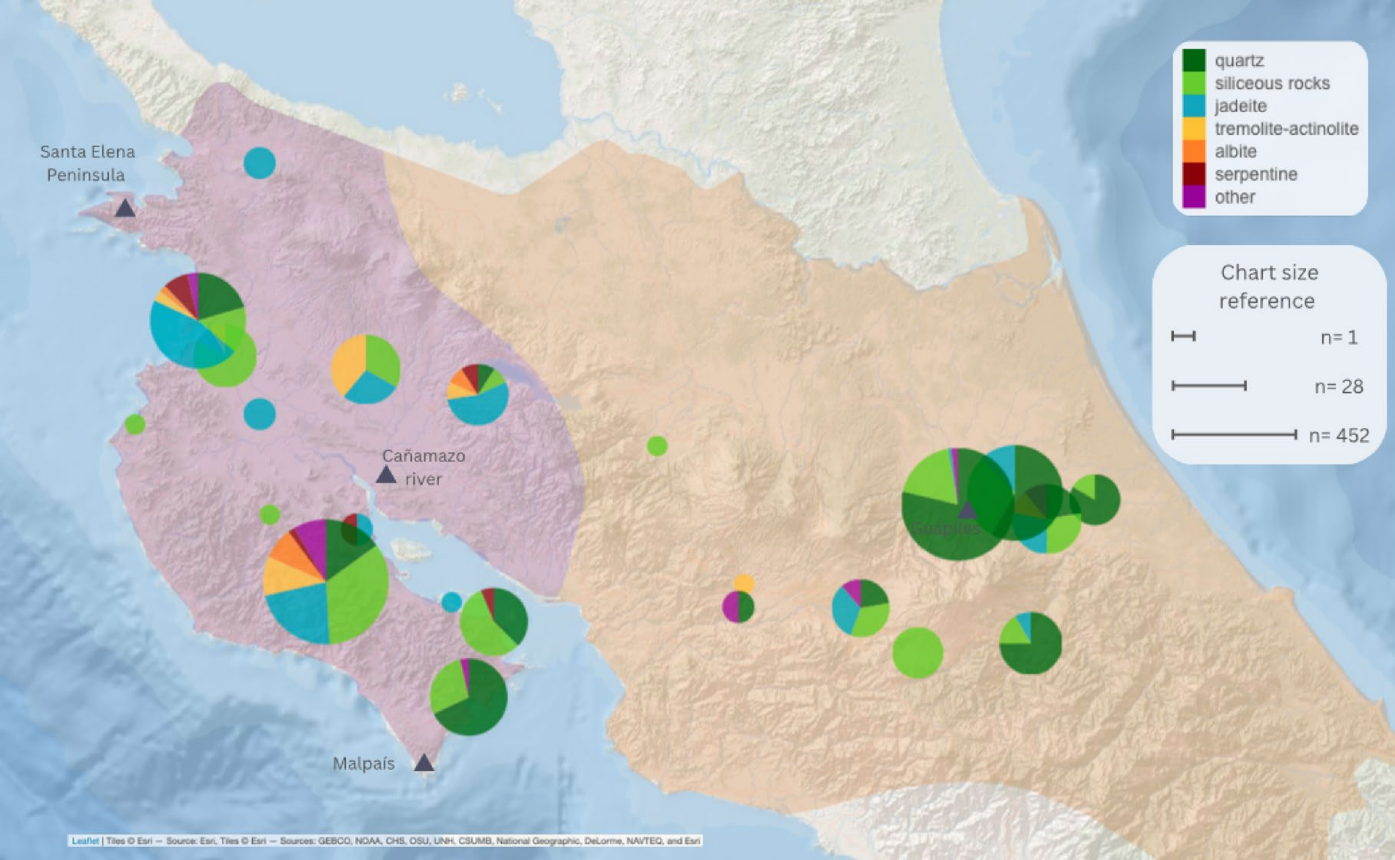
Spatial distribution of lithic artifact frequencies by archaeological region. Chart diameters reflect relative number of analyzed artifacts and were scaled using a transformation to ensure visibility of sites with strongly different number of artifacts. A scaled chart size reference includes three reference diameters and their corresponding artifact count. Archaeological regions follow the definition given in (Fig. 2b). Geographic locations mentioned in the text are indicated on the map by dark grey triangles. Base map: Esri WorldPhysical © Esri, USGS, NOAA; Esri Ocean Basemap © Esri, USGS, NOAA.

As reflected by the distribution map of analyzed material (Fig. 6), certain areas exhibit a high density of archaeological sites containing greenstone objects, crafted from heterogeneous geological materials. Within the Greater Nicoya region, an important proportion of these sites are located along the Pacific coast, notably at Culebra Bay and the inlet of the Nicoya Gulf; both of which were highly probable ports for sailors who traveled along the Pacific coast^44^. In the Central Caribbean region, a high-density area is observed near the present-day town of Guápiles, near the Atlantic slope of the mountain range that delimits the Central valley. This territory includes a series of watersheds, with major rivers flowing down from the Talamanca cordillera to the Caribbean Sea. Archaeological evidence indicates that major centers, developed over several centuries, were often established on navigable parts of the rivers. Such locations likely facilitated movement along the waterways, easing the exchanges of goods or other social interactions, possibly including long-distance voyages across the Caribbean Sea.

**Fig. 7:**
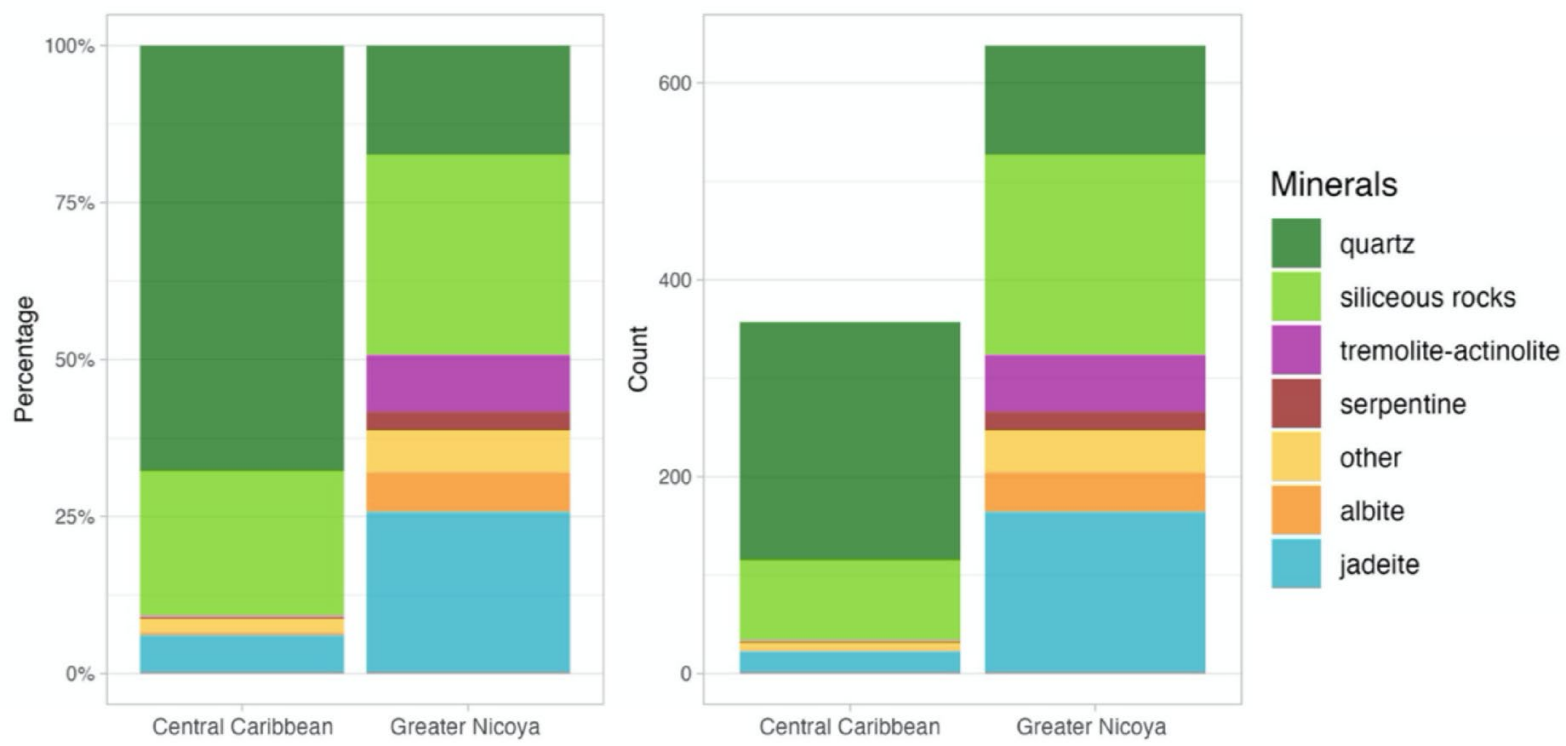
(**a**) Percentage of each mineral identified in the Central Caribbean region (left) and Greater Nicoya (right). (**b**) Number of artifacts of each mineral identified in the Central Caribbean Region (left) and Greater Nicoya (right).

A wide diversity of lithic materials was to produce greenstone artifacts, with distinctive patterns between Greater Nicoya and Central Caribbean cultural areas (Figs. 6 and 7). Indeed, the range of rocks identified in the Greater Nicoya region is considerably more diverse than in the Central Caribbean region. In particular, albitites, serpentinites, tremolite-actinolite rocks (respectively 6%, 3% and 9% of the analyzed artifact from Greater Nicoya) together with less frequently used prehnite and muscovite stones, occur almost exclusively in archaeological sites in the northern Pacific of present-day Costa Rica.

Jadeitite objects were recovered across both cultural areas with clearly distinct patterns (Fig. 7). In the Greater Nicoya region, 26% of analysed greenstone objects (163 of 638 objects) were made of jadeitite, whereas in the Central Caribbean the proportion is more than 4 times lower, at only 6% (21 of 357 objects). Such difference is also clearly denoted in the approximate volume (calculated by multiplying height, length and width of each artifact) of the jadeitite artifacts analyzed, with approximately 1420 cm^3^ of jadeitite identified in sites from the Atlantic Watershed and roughly 5060 cm^3^ in the Greater Nicoya region.

In the Central Caribbean region, the most frequently identified mineral is the quartz, present in around 67% of the samples (approximately 1400 cm^3^). In most of the cases this material has a greenish coloration and lustrous appearance that greatly resembles jadeitite. Although this type of green quartz has also been found in archaeological sites from the Greater Nicoya region, its relative abundance and volume in the Northern Pacific (around 17%, ~ 950 cm^3^) are markedly lower than in the Atlantic watershed. Siliceous rocks were also found to be widely distributed in archaeological sites in both regions, it was identified in 23% (83 of 357) of the analyzed objects from the Central Caribbean and in 32% (204 of 638) of the samples from the Greater Nicoya.

### Archaeological implications

Many of the identified materials naturally occur from local deposits in Costa Rica^45^. Alvarado and García-Casco (2019) reported possible sources of: (i) tremolite-actinolite and serpentinite rocks in the Santa Elena peninsula, (ii) quartz and siliceous rocks all over the Costa Rican territory. Regarding the quartz group (rock crystal, opal, silexite, amethyst, jasper, chalcedony, flint, etc.), the same authors reported 14 deposits in the Guanacaste region, as well as 7 others in the Atlantic watershed and in the central valley. The authors mention that in the Greater Nicoya Region different quartz varieties can be found in the Cañamazo river, as well as in several coastal areas like Malpaís and the Santa Elena Peninsula.

The widespread distribution of quartz rocks across the country explains its predominant use in local Pre-Columbian greenstone manufacturing. However, green quartz was found far more extensively in the Caribbean region (68% of the artifacts analyzed, versus 17% in Greater Nicoya), which could most likely reflect the presence of accessible deposits in the region with favorable aesthetic and mechanical properties. The group of siliceous rocks appears to be ubiquitous throughout Costa Rica. Alvarado and García-Casco (2019) notably mentioned one green quartz deposit, nearby the Guácimo river near the present-day town of Guápiles, which could be a strong candidate for a pre- Columbian exploitation. Although additional analytical data are required to reach a definitive conclusion, such deposits demonstrate that such stone was clearly available locally.

Nephrite and serpentine rocks are highly specific to the Greater Nicoya area, with local deposits on the Santa Elena Peninsula. They were only found in artefacts coming from sites within the Greater Nicoya region, with the exception of two beads from the La Fábrica and Cenada sites located to the west of the central valley. This pattern clearly indicates that the exchange networks operating between sites in these two archaeological regions (Greater Nicoya and the Caribbean) involved only selected types of greenstones, principally jadeitite and albitite. Even though the two cultural regions shared strong cultural traditions (Dios-Hacha typology, lithic industries, mace-head production, etc.) and exchanged various types of ceramics and other stone materials, particularly after 500 A.D.^46^, certain materials, such as nephrite and serpentine were not exchanged between them. Such evidence points to a sophisticated understanding of lithic resources among indigenous populations and reinforces the interpretation that raw jadeitite was intentionally selected for the manufacture of local objects, owing either to its symbolic significance or to its distinctive physical properties.

It can be hypothesized that these local materials were primarily extracted from local sources and were exchanged within the area through a down the line exchange model (Renfrew, 1977). Indeed, the energy and organizational costs required for long-distance exchanges of such abundant resources would have largely outweighed the potential benefits. Thus, the only two rock types that can be clearly identified as non-local are albitites and jadeitites.

Jadeitite was likely the most important material exchanged among the two cultural areas, Greater Nicoya and Caribbean region. It is worth noting that jadeitite was the only foreign raw material imported into the Caribbean region (accounting for up to 6% of analyzed objects) and that, in Greater Nicoya, approximately 26% of analyzed greenstones were jadeitite. Its distribution within each cultural region is clearly different. In the Caribbean region, jadeitite is clearly restricted to several ceremonial centers (Severo Ledesma, Mercocha, Las Mercedes, Río Danta, La Guaira, Canadá and Talamanca de Tibás), whereas in Greater Nicoya it is widely distributed, reaching even smaller sites.

The closest jadeitite deposits to Costa Rica are found in Guatemala (occurrences both north and south of the Motagua River Valley), the Dominican Republic (San Juan River Complex), and Cuba (Sierra del Convento)^47^. However, jadeitite reached Costa Rica both as raw blocks, such as the one discovered in the Las Huacas cemetery in the Nicoya Peninsula^48^, used to craft locally styled artifacts, and as finished or reworked objects or sometimes bearing glyphs associated with Maya elites and royalty^18^. Thus, the hypothesis of a Motagua origin remains the most plausible, even though no physico-chemical analysis has yet definitively linked Costa Rican jade to this specific deposit.

Doris Stone^49^, who surveyed looted sites throughout the Caribbean lowlands of Costa Rica, reported that jade artifacts were abundant at the Las Mercedes and Williamsburg sites in the Reventazón River basin, and at La Unión and Nuevo Corinto in the North Chirripó River basin, sites that were easily accessible by canoe from the Caribbean Sea or via its tributaries. However, distinguishing jadeitite from green quartz by visual inspection can be very challenging, and many artifacts may have been mistakenly associated with jadeitite. Moreover, the broader term “social jade” is often used in archaeology to describe all artifacts employed in social or ritual contexts, without distinction of rock type. Yet neither of these concepts is sufficient to assess the archaeological significance of interregional exchanges in the production of lapidary objects, as they do not allow the differentiation of foreign stones acquired through long-distance trade from locally sourced materials. It is therefore essential to clearly distinguish allogenic jadeitite from rocks of local origin.

It should also be noted that looting may have altered the relative proportions of different stone types, as looters preferentially targeted elite graves, which most likely contained the largest quantities of jadeitite. Nevertheless, material recovered from unlooted archaeological contexts, such as Río Danta in the Caribbean region, or Manzanillo in Greater Nicoya, corresponds well with the overall results of the project.

The albitites used for artifact manufacture in Pre-Columbian Costa Rica represent up to 4% in analyzed artefact and are clearly coming from allochthonous deposits. As no major albitite deposits have been reported in Costa Rica, it can be hypothesized that these stones were exchanged together with jadeitite. It could also have been traded through interregional exchanges, possibly by mistake or through fraudulent substitution. In Guatemala, the jadeitite deposits of the Motagua Valley are associated with the presence of albitite^50–53^. Moreover, this rock was also used as raw material for elite goods in the maya area^41,54,55^ and Teotihuacan areas^24^.

These results clearly highlight the importance of jadeitite in interregional exchanges, most likely due to its symbolic significance for Pre-Columbian cultures and its unique physical properties, particularly its hardness and color. Numerous authors^2,4,7,49^ have emphasized the importance of Costa Rica’s lithic tradition, especially in the working of greenstones. The quantity and wide distribution of jadeitite in Costa Rica provide strong evidence of the stone’s significance for Pre-Columbian societies and economies. It is therefore essential to trace the jadeitite present in Costa Rica and to determine its geological origin through geochemical analyses. This is one of the main objectives of the *MayaCosta Project*^56^, which began in 2023 and was funded by the French *Agence Nationale de la Recherche*.

## Conclusions

The results show that jadeite and albite of clear allochthonous origin, possibly extracted from the Motagua Valley in Guatemala, were more extensively distributed across the Greater Nicoya sites, whereas its occurrence in sites from the Central Caribbean region is much scarcer, suggesting a restricted model of long-distance exchange. Greater Nicoya also exhibits a higher diversity in greenstone types, including locally available materials such as serpentine and tremolite-actinolite, that were found almost exclusively in this region, near the potential extraction zone of the Santa Elena Peninsula. In contrast, quartz and other siliceous rocks, naturally abundant throughout the country, were widely distributed in archaeological sites from both regions. Overall, the spatial distribution of locally occurring materials suggests that they were obtained through short-range procurement systems. Altogether, these observations indicate that only jadeite and albite circulated through long-distance trade, while locally sourced rocks were mostly used close to their area of origin. These findings support the main objective of this study: both local and long-distance exchange networks played key and complementary roles in pre-Columbian Costa Rica; however, it was jadeite, through its circulation that seems to have bridged regional and interregional lithic traditions. Future studies should aim to determine the precise source of the jadeite that reached Costa Rica through detailed geochemical methods.

## Supplementary Information

Below is the link to the electronic supplementary material.

Supplementary Material 1

## Data Availability

Restrictions apply to the availability of these data. The archaeological specimens and associated analytical results are held in the collections of the participating museums, and access is subject to prior authorization from the relevant institutions. Consequently, the data are not publicly available but can be obtained from the corresponding author upon reasonable request and with permission from the museums.
