# Supplementary material for: Greenstone artifacts in pre-Columbian Costa Rica: from raw material to local and interregional exchanges
Source: Sci Rep. 2026 May 17;16:22339. doi: 10.1038/s41598-026-52689-z (PMC13376652; doi:10.1038/s41598-026-52689-z)
Supplement: Supplementary file 1 — Supplementary Material 1 [file 41598_2026_52689_MOESM1_ESM.docx]

Supporting information for

Greenstone artifacts in Pre-Columbian Costa Rica: from raw material to local and interregional exchanges

***Supplementary Table S1.***

***Summary of number of artifacts of each main mineral group by archaeological site***

| Archaeological Region | Archaeological site | List of artifacts analyzed^a^ | Mineralogical diversity |
| --- | --- | --- | --- |
| Greater Nicoya | Bagaces | 4416, 4443, 4447 | Jadeite= 3 |
| Greater Nicoya | Ballena | 103, 106, 107, 108, 109, 111, 112, 113, 114, 116, 118, 129, 130, 137, 144, 18, 49, 52, 55, 56, 74, 83, 84, 89, 91, 92, 93, 94 | Quartz= 19  Siliceous rocks= 8  Other= 1 |
| Greater Nicoya | Bolivar | 71, NA | Tremolite-actinolite= 1  Serpentine= 1 |
| Greater Nicoya | Delicias | 32 | Jadeite= 1 |
| Greater Nicoya | Desagüe | 127, 146, 147, 157, 20 | Siliceous rocks= 4  Tremolite-actinolite= 1 |
| Greater Nicoya | El Chilar | 136 | Siliceous rocks= 1 |
| Greater Nicoya | El Conchal | 1 | Siliceous rocks= 1 |
| Greater Nicoya | El Silo | 26, 331 | Jadeite= 1  Serpentine= 1 |
| Greater Nicoya | Finca Linares | 115, 125, 14, 15, 16, 17, 174, 18, 19, 2, 20, 9 | Siliceous rocks= 10  Jadeite= 1  Tremolite-actinolite= 1 |
| Greater Nicoya | Huiscoyol | 116A, 116E, 122A, 13, 17, 2, 3, 47, 75A, 75C, 77A, 77B, 91, 92A, 92B, 92C | Quartz= 6  Siliceous rocks= 9  Serpentine= 1 |
| Greater Nicoya | La Ceiba | 80, 137 | Jadeite= 2 |
| Greater Nicoya | La Regla | 1 | Jadeite= 1 |
| Greater Nicoya | Las Huacas | 12769_13, 12769_14, 12769_15, 12769_16, 12769_17, 12769_18, 12769_19, 12769_20, 12769_21, 12769_22, 12769_23, 12769_25, 12769_27, 12769_28, 12769_29, 12769_30, 12769_31, 12769_32, 12769_33, 2438_1010, 2438_1013, 2438_278, 2438_288, 2438_294, 2438_296, 2438_298, 2438_310, 2438_311, 2438_312, 2438_313, 2438_315, 2438_317, 2438_318, 2438_320, 2438_321, 2438_322, 2438_325, 2438_327, 2438_331, 2438_332, 2438_333, 2438_335, 2438_337, 2438_340, 2438_341, 2438_354, 2438_366, 2438_367, 2438_372, 2438_376, 2438_381, 2438_386, 2438_395, 2438_396, 2438_398, 2438_401, 2438_405, 2438_408, 2438_410, 2438_414, 2438_420, 2438_432, 2438_433, 2438_434, 2438_435, 2438_440, 2438_444, 2438_448, 2438_451, 2438_452, 2438_454, 2438_463, 2438_474, 2438_475, 2438_485, 2438_486, 2438_503, 2438_506, 2438_507, 2438_513, 2438_514, 2438_516, 2438_522, 2438_525, 2438_976, 2438_991, 2438_993, 2438_996, 2793_100, 2793_125, 2793_126, 2793_133, 2793_136, 2793_14, 2793_142, 2793_143, 2793_145, 2793_15, 2793_154, 2793_158, 2793_1585, 2793_159, 2793_160, 2793_161, 2793_162, 2793_2066, 2793_31, 2793_36, 2793_42, 2793_43, 2793_44, 2793_51, 2793_54, 2793_61, 2793_62, 2793_63, 2793_64, 2793_66, 2793_70, 2793_76, 2793_80, 2793_85, 2793_86, 2793_89, 2793_92, 2793_97, 2793_98, 2793_99, 2839_308, 2939_1028, 2939_1029, 2939_1030, 2939_1031, 2939_1033, 2939_1035, 2939_1036, 2939_1037, 2939_1038, 2939_1040, 2939_1041, 2939_1042, 2939_1043, 2939_1044, 2939_1045, 2939_1046, 2939_1047, 2939_1048, 2939_1049, 2939_1053, 2939_1059, 2939_1063, 2939_1064, 2939_1066, 2939_1068, 2939_1069, 2939_1070, 2939_1071, 2939_1072, 2939_1077, 2939_1079, 2939_1080, 2939_1081, 2939_1162, 2939_1175, 2939_1182, 2939_1197, 2939_1198, 2939_1201, 2939_1202, 2939_1203, 2939_1204, 2939_1205, 2939_1206, 2939_1207, 2939_1208, 2939_1209, 2939_1211, 2939_1212, 2939_1213, 2939_1214, 2939_1215, 2939_1216, 2939_1217, 2939_1218, 2939_1220, 2939_1221, 2939_1222, 2939_1226, 2939_1227, 2939_1229, 2939_1230, 2939_1231, 2939_1232, 2939_1233, 2939_1234, 2939_1235, 2939_1236, 2939_1237, 2939_1238, 2939_1239, 2939_1240, 2939_1241, 2939_1242, 2939_1243, 2939_1244, 2939_1245, 2939_1246, 2939_1256, 2939_1267, 2939_1270, 2939_1272, 2939_1273, 2939_1274, 2939_1275, 2939_1278, 2939_1279, 2939_1280, 2939_1288, 2939_1291, 2939_1293, 2939_1294, 2939_1299, 2939_1318, 2939_1322, 2939_1323, 2939_1329, 2939_1397, 2939_1398, 2939_1399 2939_1404, 2939_1423, 2939_1592, 2939_1593, 2939_1594, 2939_1596, 2939_1597, 2939_1621, 2939_1624, 2939_1625, 2939_1628, 2939_260, 2939_261, 2939_262, 2939_263, 2939_265, 2939_266, 2939_268, 2939_269, 2939_270, 2939_271, 2939_272, 2939_273, 2939_274, 2939_275, 2939_276, 2939_277, 2939_279, 2939_281, 2939_284, 2939_285, 2939_287, 2939_288, 2939_289, 2939_290, 2939_291, 2939_292, 2939_293, 2939_295, 2939_296, 2939_297, 2939_298, 2939_300, 2939_301, 2939_302, 2939_303, 2939_305, 2939_306, 2939_308, 2939_309, 2939_310, 2939_312, 2939_313, 2939_314, 2939_328, 2939_329, 2939_627, 2939_631, 2939_632, 2939_633, 2939_634, 2939_635, 2939_636, 2939_644, 2939_645, 2939_646, 2939_654, 2939_658, 2939_659, 2939_660, 2939_664, 2939_713, 2939_715, 2939_80, 2939_82, 2939_889, 2939_890, 2939_891, 2939_892, 2939_894, 2939_900, 2939_901, 2939_902, 2939_905, 2939_907, 2939_908, 2939_909, 2939_910, 2939_912, 2939_913, 2939_916, 2939_918, 2939_930, 2939_932, 2939_933, 2939_935, 2939_936, 2939_937, 2939_940, 2939_941, 2939_945, 2939_946, 2939_958, 2939_968, 2939_973, 45, 45_3, 2438_519, 2438_828, 2438_831, 2438_841, 2438_842, 2438_858, 2438_932, 2438_935, 2438_937, 2793_164, 2793_165, 2793_166, 2793_167, 2793_168, 2793_169, 2793_170, 2793_172, 2793_173, 2793_174, 2793_176, 2793_177, 2793_178, 2793_179, 2793_180, 2793_186, 2793_187, 2793_188, 2793_196, 2793_197, 2793_199, 2793_200, 2793_202, 2793_203_0, 2793_203_1, 2793_203_10, 2793_203_11, 2793_203_12, 2793_203_13, 2793_203_14, 2793_203_15, 2793_203_17, 2793_203_2, 2793_203_3, 2793_203_4, 2793_203_5, 2793_203_6, 2793_203_7, 2793_203_8, 2793_203_9, 2793_39, 2793_40, 2793_41, 2793_6, 2793_7, 2793_8, 2793_9, 2939_1003, 2939_1055, 2939_1153, 2939_1154, 2939_1159, 2939_1161, 2939_1163, 2939_1168, 2939_1169, 2939_1173, 2939_1178, 2939_1183, 2939_1184, 2939_1185, 2939_1189, 2939_1262, 2939_1265, 2939_1266, 2939_1307, 2939_1308, 2939_1314, 2939_1321, 2939_1400, 2939_1420, 2939_1421, 2939_24, 2939_344, 2939_400l, 2939_404, 2939_405, 2939_406, 2939_407, 2939_410, 2939_411, 2939_414, 2939_416, 2939_421, 2939_425, 2939_440, 2939_442, 2939_446, 2939_458, 2939_532, 2939_536, 2939_539, 2939_553, 2939_555, 2939_579, 2939_581, 2939_588, 2939_591, 2939_593, 2939_594, 2939_630, 2939_680, 2939_896, 2939_921, 2939_955, 1067, 1142, 1149, 1171, 1181 | Quartz= 68  Siliceous rocks= 154  Jadeite= 101  Other= 38  Tremolite-actinolite= 46  Serpentine= 8  Albite= 37 |
| Greater Nicoya | Loma Corral 3 | 111, 114, 125, 62b, 62bc, 82, 83, 84, 37, 33 | Quartz= 1  Jadeite= 8  Serpentine= 1 |
| Greater Nicoya | Los Guacales | 19, 20 | Tremolite-actinolite= 2 |
| Greater Nicoya | Mamá Inés | 1, 2 | Jadeite= 2 |
| Greater Nicoya | Manzanillo | 1314, 16455, 17246, 18001, 23212, 24942, 24950, 26070, 29496, 31304, 34361 | Quartz= 1  Siliceous rocks= 3  Other= 2  Tremolite-actinolite= 3  Serpentine= 1 |
| Greater Nicoya | Monte Sele | 101, 103, 117, 133, 138, 140, 160, 169, 171, 172, 175, 102 | Siliceous rocks= 1  Jadeite= 5  Tremolite-actinolite= 4 |
| Greater Nicoya | Nacascolo | 111, 167, 361 | Serpentine= 3 |
| Greater Nicoya | Nacascolo-Vidor | 32811, 33046, 32555, 32287, 32900, 32476, 32448, 32377, 32449, 32279, 33040, 33021, 32450, 32452, 32696, 32586, 32446, 32994, 32611, 32495, 32478, 32467, 32591, 32832, 32561, 32587, 32436, 32505, 32539, 32534, 32413, 32358, 32567, 32499,32501 32566, 32576, 32497, 32542, 32548, 32504, 32293, 32487, 32612 | Quartz= 15  Siliceous rocks= 3  Jadeite= 25  Albite= 1 |
| Greater Nicoya | Orocú | 30 | Siliceous rocks= 1 |
| Greater Nicoya | Sabana Grande | NA | Siliceous rocks= 1 |
| Greater Nicoya | Sojo | 3, 6, 7, 8, 21 | Quartz= 1  Siliceous rocks= 1  Jadeite= 2  Albite= 1 |
| Greater Nicoya | Vidor | 1115, 1167, 2518, 2998 | Siliceous rocks= 4 |
| Greater Nicoya | Vistas del Coco | 129A, 129B, 129C, 129D, 129E, 129F, 129G, 151, 281, 295, 464, 465, 660, 488 | Siliceous rocks= 3  Jadeite= 5  Other= 1  Serpentine= 2  Albite= 1 |
| Central Caribbean | Azul | 3, 4, 6, 8 | Quartz= 3  Siliceous rocks= 1 |
| Central Caribbean | Bremen_b | 122, 152, 153, 173a, 173d, 173f, 180, 56 | Quartz= 4  Siliceous rocks= 4 |
| Central Caribbean | CENADA | 2, 3, 16 | Siliceous rocks= 2  Other= 1 |
| Central Caribbean | Campus | 20, 67 | Albite= 2 |
| Central Caribbean | Canadá | 158, 378, 379 | Siliceous rocks= 2  Jadeite= 1 |
| Central Caribbean | Carlos Aguilar Piedra | 86, 89 | Siliceous rocks= 2 |
| Central Caribbean | Hacienda el Molino | 37, NA | Siliceous rocks= 2 |
| Central Caribbean | La Cabaña | 2, 3 | Quartz= 2 |
| Central Caribbean | La Fábrica | 160 | Tremolite-actinolite= 1 |
| Central Caribbean | La Fuente | 13, 37 | Quartz= 1  Siliceous rocks= 1 |
| Central Caribbean | La Guaira | 18h, 18n, 98, 100, 102, 101, 104, 137c, 137e, 143, 142, 75, 74, 73, 47, 40, 128, 127, 126, 125, 124, 123, 122, 121, 120, 119, 118, 117, 116, 114, 112, 111, 110, 135, 133, 131, 130, 18p, 18j, 18d | Quartz= 33  Siliceous rocks= 4  Jadeite= 3 |
| Central Caribbean | La Montaña | 17, 18 | Quartz= 1  Siliceous rocks= 1 |
| Central Caribbean | Las Mercedes | 35_559, 35_548, 35_567a_d | Siliceous rocks= 1  Jadeite= 2 |
| Central Caribbean | Liceo | 116, 131 | Siliceous rocks= 2 |
| Central Caribbean | Los Sitios | 339 | Quartz= 1 |
| Central Caribbean | Mercocha | 068-016-65, 068-014-65, 068-009-65, 068-020-65, 068-018-65 | Jadeite= 3  Other= 2 |
| Central Caribbean | Pan de Azúcar | 191, 90 | Quartz= 1  Other= 1 |
| Central Caribbean | Playa Hermosa | 319 | Quartz= 1 |
| Central Caribbean | Polideportivo-b | 11, 318, 326, 4, 6, 96 | Quartz= 5  Siliceous rocks= 1 |
| Central Caribbean | Río Danta | 101a, 101c, 102c, 105, 108, 110, 110a, 120, 130a, 130b, 130c, 130cpeq, 137a, 137e, 139, 137d, 137c, 137b, 137f, 137g, 145l, 146, 158, 170, 175, 194, 195, 197, 20, 201, 21, 22, 222f, 23, 231, 231b, 233, 235, 236, 237a, 237b, 237c, 237cu, 237d, 237e, 237f, 24, 241, 243, 25, 253, 254, 255, 256, 257, 258, 26, 268cu1, 268cu2, 27, 270, 28, 280, 29, 3, 318 a, 318 b, 318 c, 318 d, 318, 32 33, 331, 332, 34, 343, 344 a, 344 b, 35, 358, 359, 36, 367, 367 a, 367 b, 367 c, 368 a, 368 b, 368 c, 368 d, 368 e, 37, 382 a, 382 b, 385 a, 385 b, 385 c, 385 d, 385, 393a, 393b, 393c, 393d, 396, 399, 401, 427, 434, 435, 436, 437, 438, 442, 451, 468, 474, 475, 476, 477, 486, 487, 489 a, 489 b, 489, 489g, 489 f, 497 a, 497, 507, 510, 512,519a, 519 b, 519a, 519, 529, 529 e, 537 a, 537, 539, 547, 548, 552, 559, 565 a, 573, 575 a, 579, 579, 579, 584, 584, 584_f, 584, 584, 59, 608 a, 608, 608 e, 612, 614, 617, 636, 667 c, 670, 69, 694, 70, 700 a, 72, 720 b, 723 b, 723, 73, 737, 754, 76, 762, 777, 784, 785, 79, 80, 92, 93, 94, 95, 96, 97, 344_tabp, 344_tvg, 381, 474, 474_2, 489, 497, 529, 537, 565, 579, 584, 608, 667, 700, 720, 762, 771, 777, 784 | Quartz= 172  Siliceous rocks= 38  Jadeite= 2  Other= 4 |
| Central Caribbean | Severo Ledesma | 16, 195, 4, 63, 64, 65, 66, 67, 70, 72, 73, 75, 76, 77, 78, 80, 84, 86, 89, 93, 96, T2A7, T6A5, T6A6, T6A7, T6A8, 68, 45, 101, t16a1, t5a2, 26, 24, 1 | Quartz= 12  Siliceous rocks= 20  Jadeite= 7 |
| Central Caribbean | Talamanca de Tibás | 36, 19, 40, 34, 78 | Quartz= 1  Siliceous rocks= 1  Jadeite= 3 |
| Central Caribbean | Tatiscú | 23 | Siliceous rocks= 1 |
| Central Caribbean | Tres Ríos | 138, NA | Siliceous rocks= 2 |
| Central Caribbean | Valldeperas | 28 | Quartz= 1 |
| Central Caribbean | Zapote-2 | 25, 26 | Quartz= 2 |

^a.^This column includes the list of artifacts analyzed per site. The format of the artifact identifier varies according to the guidelines of the collection and corresponding Museum.

***Supplementary Table S2.***

***Summary of FTIR and XRD results of less frequent material groups identified***

| Group | Infrared spectroscopy | | XRD |
| --- | --- | --- | --- |
|  | Number of samples | Frequency and assignment^1–3^ |  |
| Mica^a^ | 14 | 1040 cm^-1^ Si-O stretching  920 cm^-1^  coordinated OH bending  530 cm^-1^ Si-O-Al vibrations  477 cm^-1^ Si-O-Si bending | Identification of muscovite by analysis of sample 2939-558 |
| Feldspars^b^ | 8 | 1140 cm^-1^ Si-O stretching  1045, 1010 cm^-1^ Si(Al)-O stretching  640, 609, 584 cm^-1^ O-Si(Al)-O bending  538, 466 cm^-1^ Coupling between O-Si-O bending and M-O stretch  433 cm^-1^ Si-O-Si deformation | Not analyzed by XRD |
| Chlorite^a^ | 4 | 1250-400 cm^-1^ OH bending  550-400 cm^-1^ (Fe,Mg)-O-Si or Al-O-Si bending | Not analyzed by XRD |
| Prehnite | 2 | 1072, 991, 938 cm^-1^ Al-O-Si stretching vibration  814 cm^-1^ H-O-Fe bending vibration  532, 472 and 419 cm^-1^ Si-O-Al bending vibration | Not analyzed by XRD |

^a.^ Through the infrared spectra obtained it was not possible to identify the specific mineral of this group because of the spectral resolution limitations intrinsic to the FTIR reflection mode

^b.^ Their spectra show clear differences with respect to albite and therefore they are not assigned as such, however they have bands characteristic of other end-members like k-feldspar and plagioclase. Because of the similarity of the end-member spectra and the apparent mixed mineralogical composition of these rocks, a clearer distinction cannot be made.

***Supplementary Material S3.***

***Details of the principal component analysis (PCA).***

A series of pre-processing procedures were applied to the spectroscopic data using R software and were chosen according to the spectral requirements: computing of mean spectra per sample, cropping (the range of the FT-IR spectra was cropped to 400–1500 cm^-1^), Kubelka-Munk transformation to reduce influence of scattering effects, rubberband baseline correction, normalization of spectrum intensities and Savitzky-Golay smoothing interpolation. Statistical analysis and data pre-processing was performed using the computing environment R. The PCA was done using the *prcomp* function from the *stats* package, which is core part of the R standard library in R.

Principal Component Analysis (PCA) is a widely used statistical technique for reducing the dimensionality of complex datasets while preserving most of the original information. It allows to summarize large sets of variables into a smaller number of components, reveal underlying patterns and correlations within the data, and facilitate visualization in lower-dimensional spaces. PCA works by transforming the original variables into new, uncorrelated variables called principal components, which are linear combinations that maximize the explained variance.

Principal Components 1 and 2 are presented in Figure 3 of the publication. As shown in Figure S3-1, they account for 72.5% of the total variance of the dataset.


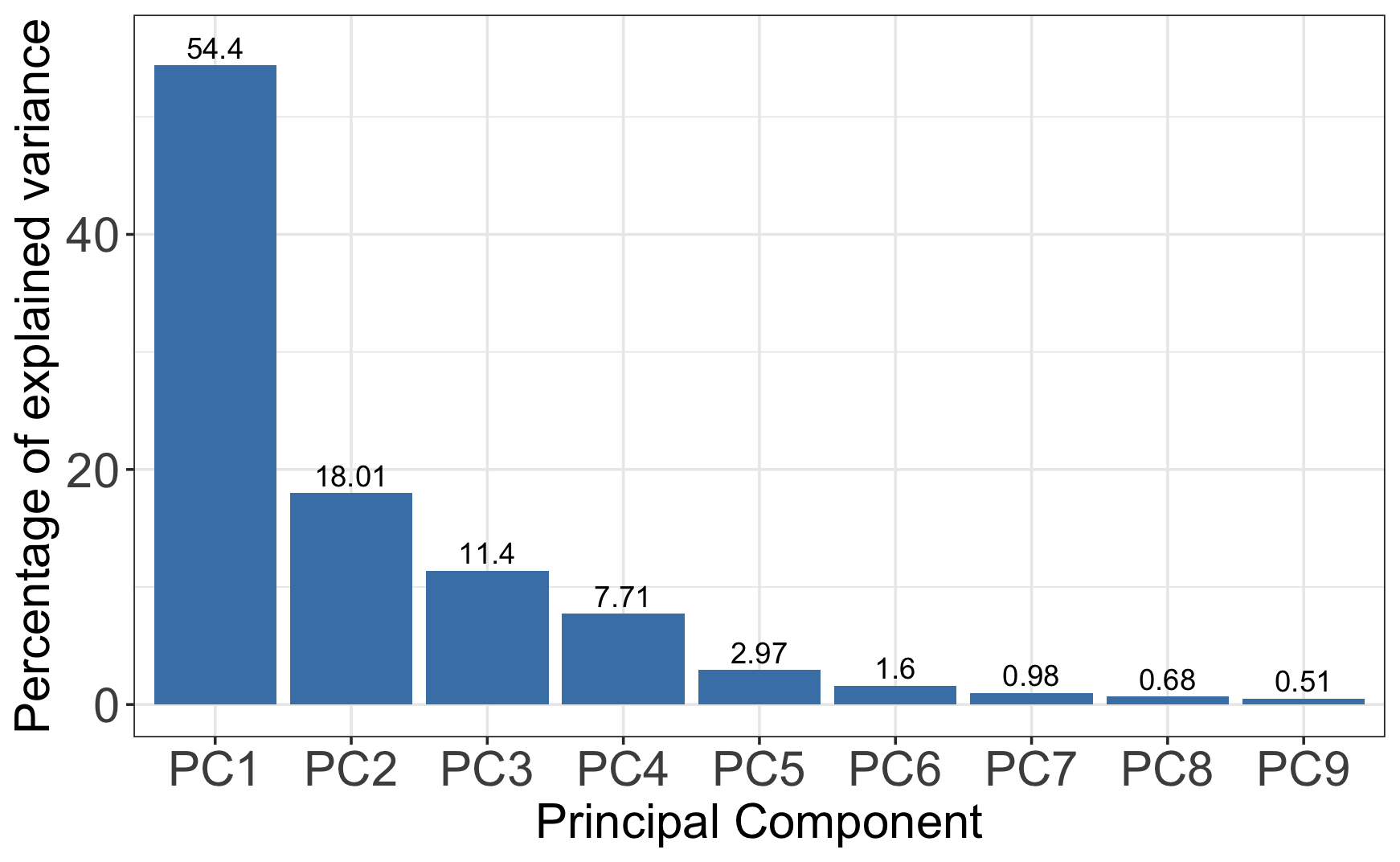


*Figure S3-1 : Percentage of variance explained by Principal components.*

The loading spectra are presented in Figure S3-2.


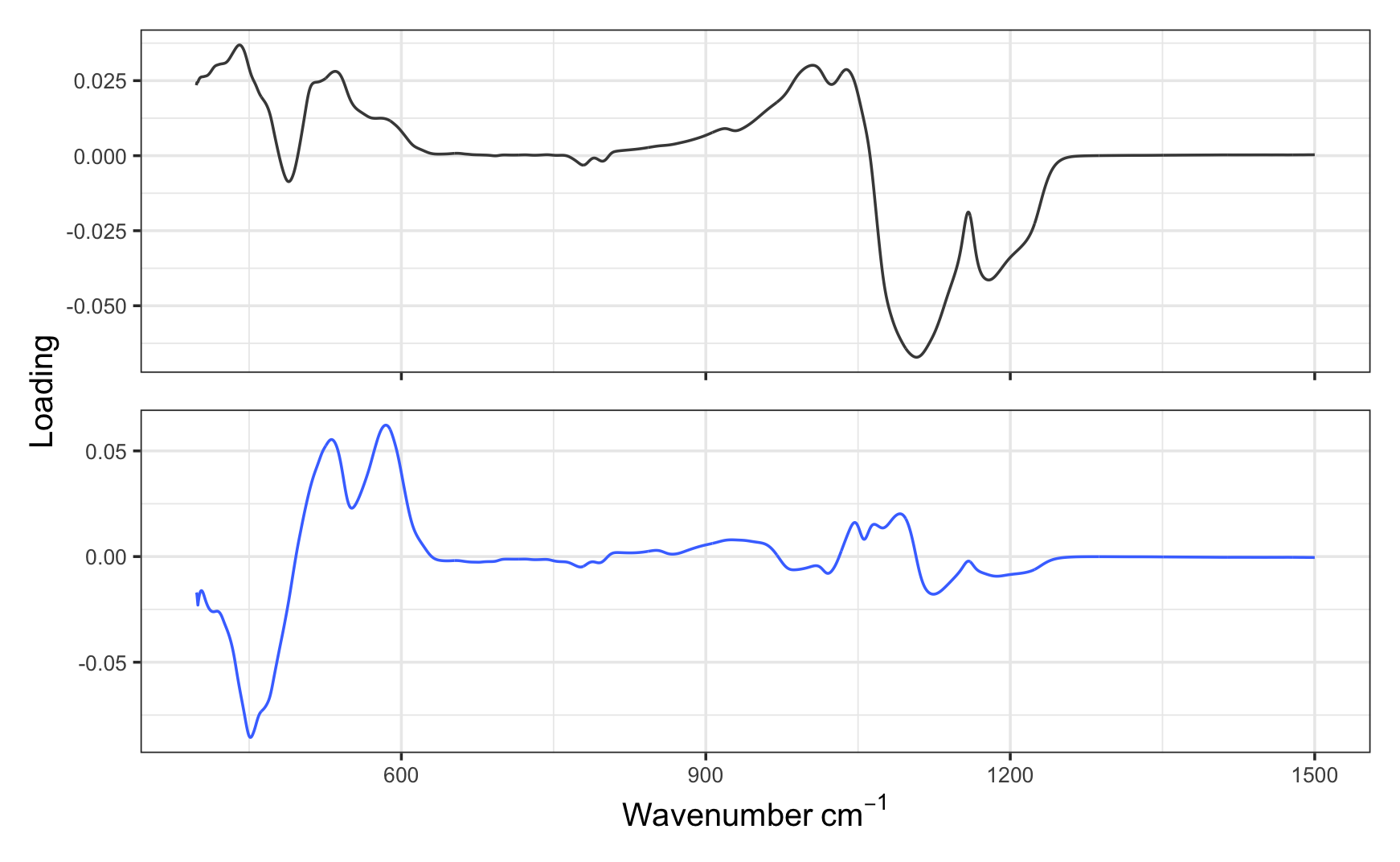


*Figure S3-2. Loading spectra of Principal Components 1 and 2.*

The following loading maxima are observed in Figure S3-2. Letters indicate relative intensity (ww = weak, m = medium, s = strong), and mineral assignments are provided where applicable:

- PC1: positive bands at approximately 441 (m, jadeite), 536 (m, tremolite-actinolite), 1003 (m, albite), and 1037 (m, albite), and negative broad bands at approximately 1107 (s, quartz) and 1179 (quartz).
- PC2: positive bands at approximately 531 (s, jadeite), 585 (s), 1046 (m, albite), 1065 (m, tremolite-actinolite), 1093 (m), and 1159 (m, albite), and negative bands at approximately 451 (s, tremolite-actinolite) and 1125 (m).

As a result, we can show that PC1 is discriminating, from high to low value, quartz to siliceous rocks and other minerals (albite, tremolite-actinolite and jadeite). PC2 is discriminating, from high to low value, jadeite, tremolite-actinolite and albite, and other minerals (serpentine, quartz, siliceous rocks).

Principal Components 1 and 2 are presented in Figure 3 of the publication. As shown in Figure S3-1, they account for 72.5% of the total variance of the dataset.

Figure S3-1. Percentage of variance explained by principal components.

The loading spectra are presented in Figure S3-2.

Figure S3-2. Loading spectra of Principal Components 1 and 2.

The following loading maxima are observed in Figure S3-2. Letters indicate relative intensity (ww = weak, m = medium, s = strong), and mineral assignments are provided where applicable:

* PC1: positive bands at approximately 441 (m, jadeite), 536 (m, tremolite–actinolite), 1003 (m, albite), and 1037 (m, albite), and negative broad bands at approximately 1107 (s, quartz/siliceous rocks) and 1179 (quartz).

* PC2: positive bands at approximately 531 (s, jadeite), 585 (s), 1046 (m, albite), 1065 (m, tremolite–actinolite), 1093 (m), and 1159 (m, albite), and negative bands at approximately 451 (s, tremolite–actinolite) and 1125 (m).

Overall, PC1 discriminates, from high to low values, quartz and siliceous rocks from other minerals (albite, tremolite–actinolite, and jadeite). PC2 discriminates, from high to low values, jadeite, tremolite–actinolite, and albite from other minerals (serpentine, quartz, and siliceous rocks).

⸻

If you want, I can make it more concise or adapt it to a specific journal style (Elsevier, ACS, etc.).

***References (Supplementary Information)***

1. Yang, M. *et al.* Near-Infrared Spectroscopic Study of Chlorite Minerals. *J. Spectrosc.* **2018**, 6958260 (2018).

2. Wang, Q., Guo, Q., Li, N., Cui, L. & Liao, L. Study of the mechanism of color change of prehnite after heat treatment. *RSC Adv.* **12**, 3044–3054 (2022).

3. Sayin, M. & Graf von Reichenbach, H. Infrared spectra of muscovites as affected by chemical composition, heating and particle size. *Clay Miner.* **13**, 241–254 (1978).
